# Anticoking Effect of Eu^3+^ Doping of the Ru/Ceria Catalyst in the MSR Reaction for Hydrogen Generation

**DOI:** 10.1021/acs.jpcc.5c05324

**Published:** 2025-10-03

**Authors:** Oleksii Bezkrovnyi, Núria J. Divins, Isabel Serrano, Xènia Garcia, Piotr Kraszkiewicz, Maciej Ptak, Mirosława Pawlyta, Leszek Kępiński, Jordi Llorca

**Affiliations:** † W. Trzebiatowski Institute of Low Temperature and Structure Research, 215275 Polish Academy of Sciences, 50-422 Wroclaw, Poland; ‡ Institute of Energy Technologies, 16767 Universitat Politècnica de Catalunya, EEBE, 08019 Barcelona, Spain; § Department of Chemical Engineering and Center for Research in Multiscale Science and Engineering, Universitat Politècnica de Catalunya, EEBE, 08019 Barcelona, Spain; ∥ School of Chemistry, University of St. Andrews, St Andrews KY16 9ST, United Kingdom; ⊥ Materials Research Laboratory, 49569 Silesian University of Technology,Gliwice 44-100, Poland

## Abstract

The positive effect of Eu^3+^ doping on the stability of the Ru/ceria catalyst during the methane steam reforming (MSR) reaction, which was used for H_2_ production, was observed. The effect is attributed to a significant inhibition of coking-induced deactivation of the catalyst by Eu doping, which we explain by three hypotheses. The first one is an increase in basicity with Eu doping, which inhibits carbon deposition on the working catalyst during the MSR reaction. The second one is that Eu addition introduces strain into the ceria lattice, which could facilitate oxygen diffusion and, as a consequence, prevents catalyst’s coking. The third one is related to the presence of an additional high-temperature pathway for supplying lattice oxygen based on Eu^3+^ → Eu^2+^ reduction on the surface of the Eu-doped ceria support.

## Graphical abstract

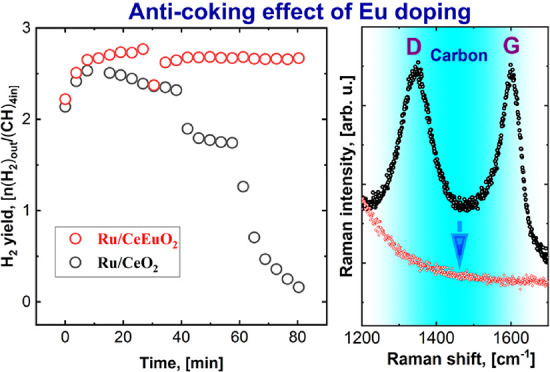

## Introduction

Coking is one of the major reasons for catalyst deactivation. Using “active supports” instead of nonreducible “inert” ones was found to inhibit catalyst’s coking.^1^ In the last decade, in addition to metals, reducible metal oxides such as CeO_2_ have also proven effective in stabilizing Ni particles against sintering at high temperatures.^2^ The presence of CeO_2_ in Ni–CeO_2_–Al_2_O_3_ catalysts has been shown to enhance methane conversion and stability, with the optimal CeO_2_ promoter content depending on the Ni loading. For instance, in a 13 wt % Ni/Al_2_O_3_ system, addition of 1.02 wt % Ce exhibited the most effective promotion, achieving a sustained 75% CH_4_ conversion at a S/C ratio of 2.7 over 300 h.^3^ Noble metal catalysts, such as Rh, Ru, Pt, Pd, and Ir, generally exhibit catalytic activity and stability higher than those of Ni catalysts. However, their widespread application is restricted by their high cost. Additionally, some noble metals are prone to aggregation and carbon deposition. Consequently, extensive research has been conducted to enhance the performance of noble metal catalysts in the steam reforming of methane while minimizing their loading amounts.

In the present study, we focus on Ru/ceria-based catalysts, which combine a relatively low price and high activity in a wide range of catalytic processes, such as C_3_H_8_ oxidation,^4^ CO_2_ methanation,^5^ and methane steam reforming (MSR).^6^ As a test reaction, we chose MSR due to its high relevance for developing hydrogen production technologies, an actively growing field of modern science. Main attention was paid to the coking resistance of Ru/ceria-based materials, a critical parameter influencing catalyst performance.

Ceria (CeO_2_) is considered a reducible “active support,” recognized for its high oxygen storage capacity based on reversible Ce^4+^ ↔ Ce^3+^ transitions, enhancing the mobility of surface oxygen ions.^7,8^ It promotes the transfer of activated oxygen from H_2_O and CO_2_ to the catalyst surface, where the coke is gasified into CO and CO_2_.^9^ We expect that doping of ceria support with Eu^3+^ ions, which have a much higher reduction temperature (>400 °C) than Ce^4+^ (20–200 °C),^10–17^ can extend its anticoking effect to the typical temperature window of the MSR reaction (400–800 °C).^18–21^ Moreover, Eu doping of the ceria support could facilitate its oxygen diffusion ability by generating additional oxygen vacancies and introducing lattice strain, thereby preventing catalyst coking. Some facilitation of the Ce_1–*x*_Eu_*x*_O_2_ reducibility was observed in our previous work.^15^ It should also be noted that due to the higher basicity of Eu_2_O_3_ than CeO_2_,^22^ the substitution of Ce^4+^ ions with Eu^3+^ in ceria support is expected to increase its basicity. Since a number of studies indicate that managing the catalyst’s basicity allows for the inhibition of its coking,^23–25^ Eu doping should improve the coke resistivity of the Ru/Ce_1–*x*_Eu_*x*_O_2_ catalyst. This, in turn, could positively impact the catalyst’s stability. Thus, in the present study, we investigated the effect of Eu^3+^ doping on the coke resistance of the Ru/CeO_2_ catalyst during the MSR reaction for hydrogen production.

## Experiments

Ru/CeO_2_ and Ru/Ce_0.80_Eu_0.20_O_2_ samples were synthesized using the wet chemistry method described previously (see details in Supporting Information).^16,26,27^ The crystal structure of the samples was determined by powder X-ray diffraction (XRD) using an X’Pert PRO PANalytical diffractometer with Cu Kα radiation. The morphology of the samples was determined by transmission electron microscopy (a probe-corrected FEI TITAN microscope operating at 300 kV and a Philips CM-20 SuperTwin instrument operating at 160 kV). Raman spectra (100–4000 cm^–1^) were measured by using a Renishaw InVia Raman spectrometer equipped with a confocal DM 2500 Leica optical microscope. The chemical composition of the samples was verified by energy-dispersive X-ray spectroscopy (EDS) using an EDAX Genesis XM4 spectrometer installed on a FEI NovaNanoSEM 230 microscope. The surface of the catalysts was studied by X-ray photoelectron spectroscopy (XPS) using a laboratory SPECS system with a PHOIBOS 150 EP Hemispherical Energy Analyzer and an MCD-9 detector. The temperature-programmed reduction (H_2_-TPR) and temperature-programmed desorption of carbon dioxide (CO_2_-TPD) were studied using Micromeritics AutoChem II 2920 equipment. The catalytic activity of the samples was tested in the MSR reaction, with the reaction products analyzed using micro-GC (Agilent Technologies 3000A Micro-GC). Additional experimental details are provided in the Supporting Information.

## Results and Discussion

Two samples were studied: undoped Ru/CeO_2_ and Eu-doped Ru/CeEuO_2_. As seen in the STEM images (Figure 1), both samples contain ceria particles decorated with Ru NPs, a few nanometers in size. Element mapping shows that almost all ruthenium is localized within the Ru nanoparticles; the Ru-related signal from the ceria support is negligible, indicating the practical absence of Ru dissolution into the ceria support (see insets in Figure 1).

**Figure 1:**
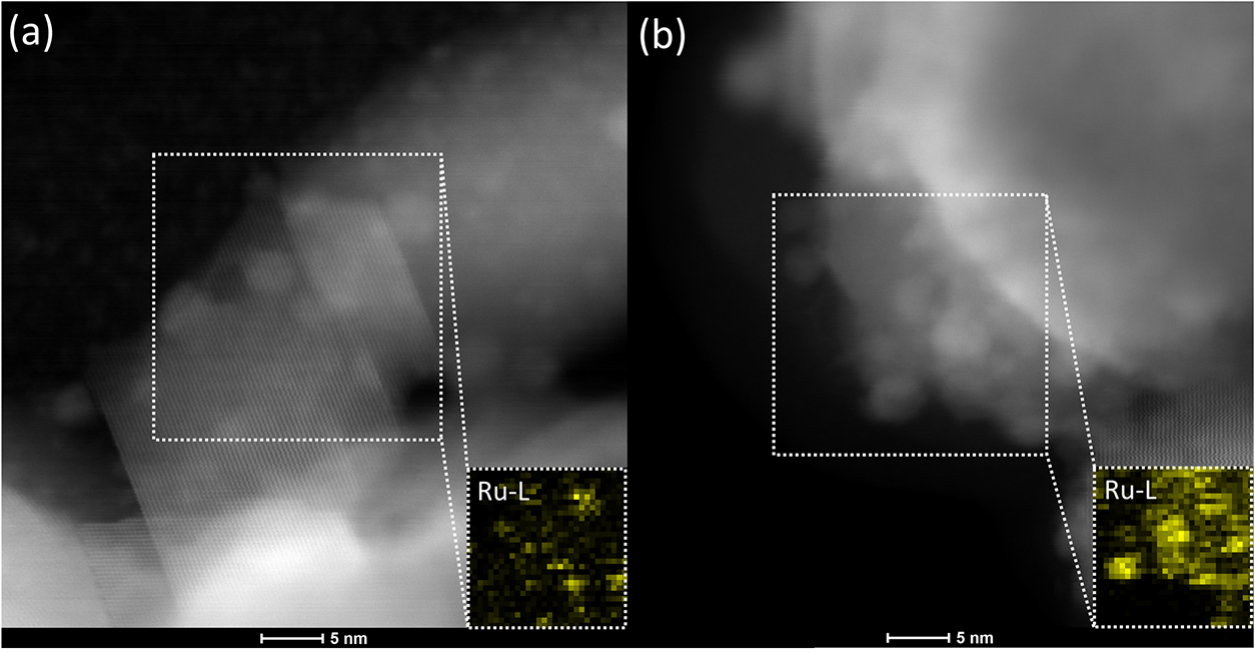
Representative HAADF-STEM images of the as-prepared Ru/CeO_2_ (a) and Ru/CeEuO_2_ samples (b). In the insets, ruthenium distribution EDS maps are shown.

The actual europium content in the Ru/CeEuO_2_ catalyst, measured by EDS, agrees with the nominal content set by the Eu concentration in the precursor solution (see Table S1). The nominal Ru content in both samples was the same, 2.5 wt %, which agrees well with the Ru contents measured by EDS (see Table S1). The powder XRD patterns of Ru/CeO_2_ and Ru/CeEuO_2_ showed only reflections corresponding to the fluorite-type CeO_2_ structure with space group *Fm*3*m* (Figure S1 and Table S2). The calculated mean crystallite sizes of the ceria support are comparable for Ru/CeO_2_ and Ru/CeEuO_2_: 31 and 36 nm, respectively. However, as seen in Table S2, Eu doping noticeably increases the lattice strain in the ceria support. A magnified part of the patterns, featuring a (111) peak, reveals its shift to a lower 2Θ angle for the Ru/CeEuO_2_ sample (Figure S1b). This shift corresponds to an expansion of the lattice parameter of the ceria substrate from 0.5413 nm for Ru/CeO_2_ to 0.5422 nm for Ru/CeEuO_2_ due to the substitution of smaller Ce^4+^ ions (0.097 nm) with larger Eu^3+^ ions (0.107 nm). No peaks related to metallic ruthenium or ruthenium oxides were detected. This result is attributed to the small particle size of Ru nanoparticles (Figures 1 and S2).

The reducibility of the as-prepared Ru/CeO_2_ and Ru/CeEuO_2_ catalysts was investigated by H_2_-TPR (see Figure S3 and Table S3). Table S3 compares the measured amounts of hydrogen consumed during H_2_-TPR and the theoretical amount of hydrogen needed for the complete reduction of RuO_2_ and the catalyst support, assuming that the catalysts were completely oxidized. It should be remembered that both samples were heated in hydrogen at 700 °C during the preparation stage and then exposed to air. Therefore, it should be assumed that the materials have been only partially oxidized, which explains the H_2_ consumption being lower than expected for fully oxidized samples (Table S3). This assumption agrees with XPS data (cf. Figure S4) and our previous work, where we demonstrated, using the NAP-XPS technique, that Ru nanoparticles are easily oxidized when exposed to an oxidative medium, even at room temperature.^27^ It should be noted that H_2_ consumption in the temperature range of 100–600 °C for Ru/CeEuO_2_ is higher than that for Ru/CeO_2_ (Table S3). This indicates that Eu doping modifies the ceria structure, shifting the bulk reducibility peak to lower temperatures. Such a shift suggests easier oxygen diffusion, which is crucial for the removal of carbon from the catalyst surface.

The catalytic activity of Ru/CeO_2_ and Ru/CeEuO_2_ was studied in the MSR reaction for H_2_ production (details in the Supporting Information). Figure 2 shows the methane conversion and H_2_ yields for the two catalysts as a function of the reaction time. The methane conversion and hydrogen yield are calculated as $X_{{\mathrm{CH}}_{4}}=\frac{{ṅ}_{{\mathrm{CH}}_{4},\mathrm{in}}-{ṅ}_{{\mathrm{CH}}_{4},\mathrm{out}}}{{ṅ}_{{\mathrm{CH}}_{4},\mathrm{in}}};,Y_{H_{2}}=\frac{{ṅ}_{H_{2},\mathrm{out}}}{{ṅ}_{{\mathrm{CH}}_{4},\mathrm{in}}}$.

**Figure 2:**
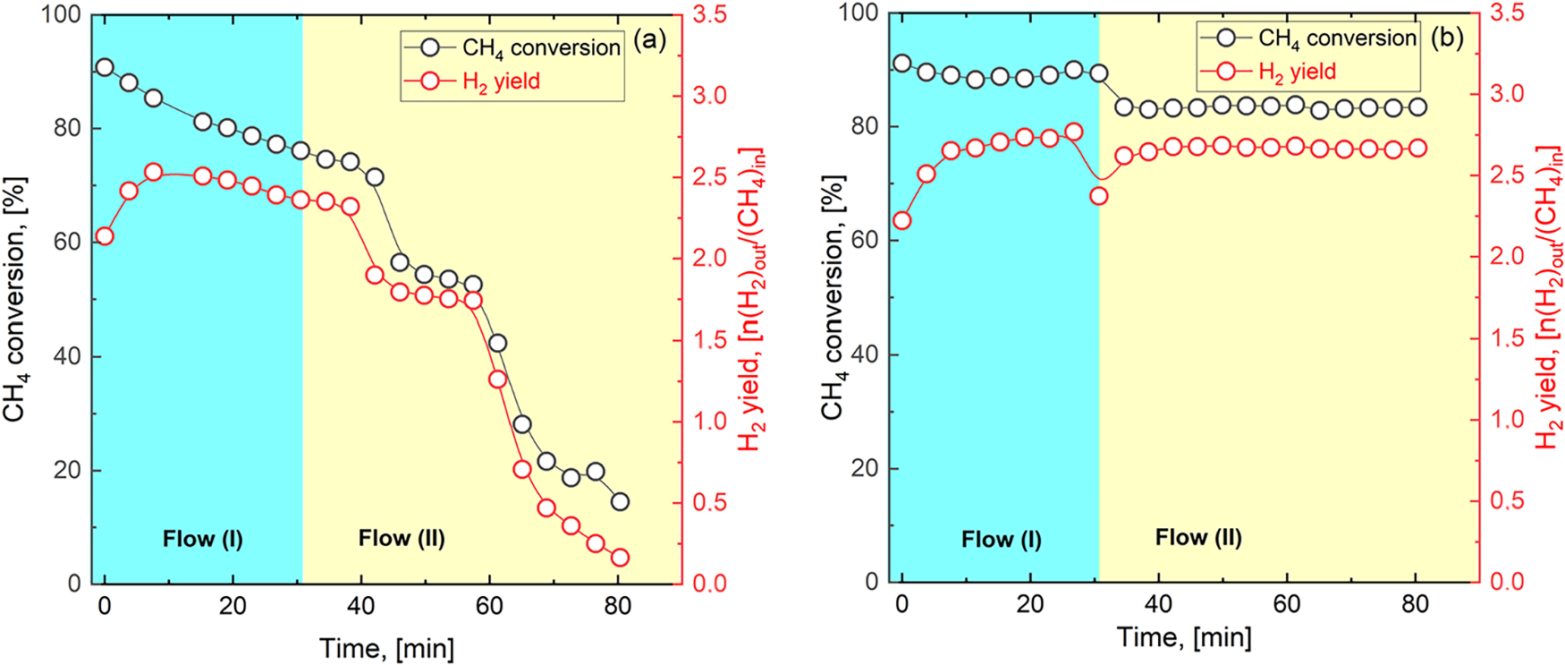
CH_4_ conversion and H_2_-production plots for the MSR reaction at 700 °C over Ru/CeO_2_ (a) and Ru/CeEuO_2_ (b) catalysts. (Flow (I): 30 mL/min N_2_, 20 mL/min CH_4_, and 40 mL/min H_2_O; Flow (II): 60 mL/min N_2_, 40 mL/min CH_4_, and 80 mL/min H_2_O).

As shown in Figure 2, the initial methane conversion over both catalysts ranged from 80% to 90%. However, as the reaction time increases, differences between nondoped and doped catalysts begin to emerge. In particular, the Ru/CeEuO_2_ catalyst appears to be much more stable (Figure 2b) than Ru/CeO_2_ (Figure 2a), especially at higher gas flow rates.

To gain a deeper understanding of how the Eu addition improves the catalyst’s stability, we analyzed Raman spectra of the undoped and Eu-doped catalysts before and after the MSR reaction (Figure 3). Before MSR, both spectra exhibit strong bands at 459 and 455 cm^–1^, corresponding to the first-order F_2g_ band of the fluorite structure, and a few low-intensity bands at about 235, 590, 695, 970, and 1155 cm^–1^ associated with the 2TA and 2TO (also referred to ceria defect band D) modes, the Ru–O–Ce bonds, and the 2TO and 2LO modes, respectively.^28,29^ The F_2g_ band of Ru/CeEuO_2_ is noticeably broader than that of Ru/CeO_2_ due to some ceria lattice disorder caused by Eu doping. It agrees with the increased lattice strain measured by XRD (Table S2).

**Figure 3:**
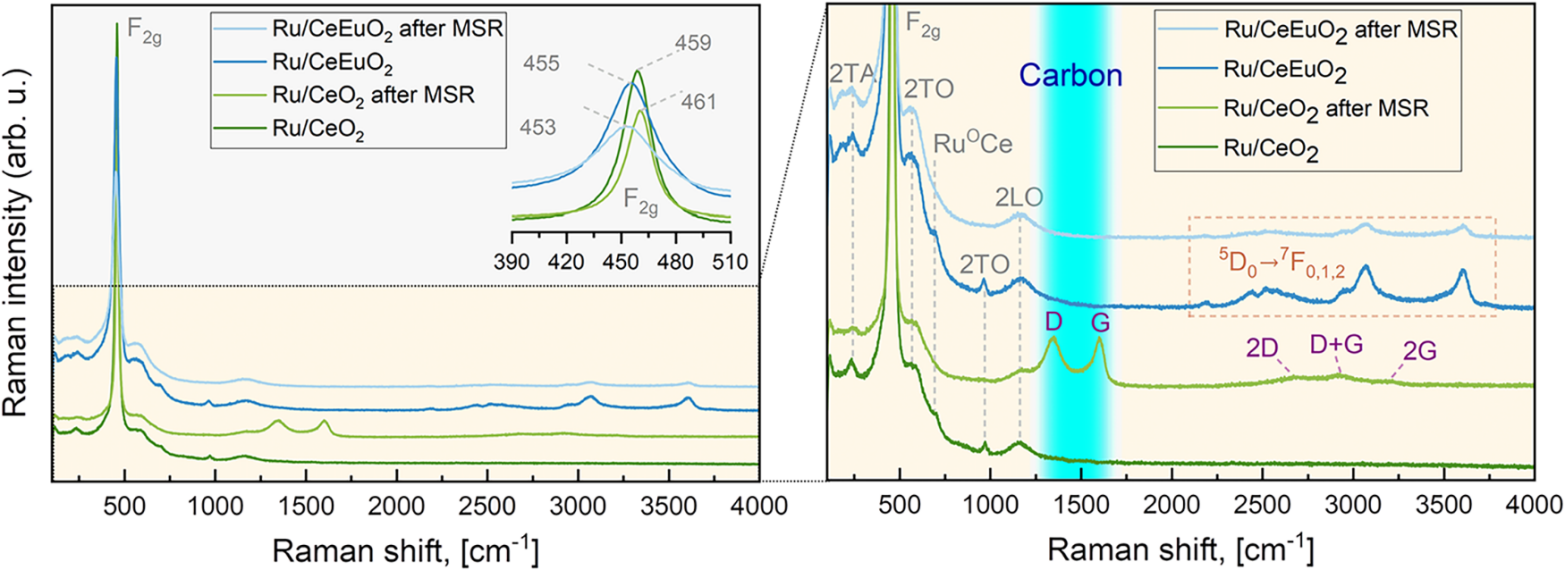
Raman spectra of the undoped (Ru/CeO_2_) and Eu-doped (Ru/CeEuO_2_) catalysts before and after the MSR reaction (inset: magnified F_2g_ region).

After MSR, the F_2g_ bands are shifted, less intense, and broadened, while the Ru–O–Ce and 2TO bands disappear, indicating higher disorder and/or an increased concentration of defects. In addition, the undoped Ru/CeO_2_ catalyst exhibits additional broad bands typical for carbon materials, observed at about 1345, 1602, 2690, 2925, and 3170 cm^–1^ and assigned to carbon defect band D, the primary mode of materials composed of a graphitic-related structure (G), D-band overtone (2D), G+D combination, and the overtone of G (2G), respectively.^30,31^ The relatively high intensity of the D-band indicates a high concentration of an amorphous structure in the deposited carbon product. The absence of D and G bands in the Raman spectrum recorded for Ru/CeEuO_2_ after the MSR process proves that the presence of Eu^3+^ ions changes the catalyst’s performance, drastically suppressing the formation of carbon byproducts. Additional contours observed for Ru/CeEuO_2_ in the 2190–3600 cm^–1^ range appear due to the characteristic ^5^D_0_→^7^F_0,1,2_ emission bands of Eu^3+^ ions excited by a 514.5 nm laser.^30^

The results of Raman spectroscopy agree well with the TEM characterization. As shown in Figure S2a–d, the amount of carbon deposited on Ru/CeO_2_ catalysts after the MSR reaction is significantly greater than that on the Ru/CeEuO_2_. Meanwhile, analysis of the C 1s + Ru 3d region of the XPS spectra of both samples reveals strong carbon contamination (Figure S4). The C-related XPS signal does not change noticeably after the MSR reaction for the Ru/CeO_2_ sample; however, it decreases strongly for Ru/CeEuO_2_. It should be noted that some apparent inconsistency between Raman and XPS data could be explained by the difference in probing depth. Raman spectroscopy is considered a “bulk technique” because the depth of the signal collection is approximately a few micrometers—much higher than the size of Ru/ceria particles. In contrast, XPS is highly surface-sensitive, collecting the signal from depths of only a few nanometers. Therefore, even a small amount of carbon contamination on the surfaces of both as-prepared Ru/CeO_2_ and Ru/CeEuO_2_ samples could be the source of a strong XPS signal. However, a strong C-related Raman signal requires much higher total carbon concentration in the sample. Thus, we can tentatively assume that despite the presence of the surface C impurities in both as-prepared samples (XPS data), Raman spectroscopy indicates that the total amount of C-contamination after the MSR reaction increases much more strongly in the Ru/CeO_2_ catalyst than in Ru/CeEuO_2_.

We propose three nonmutually exclusive hypotheses to explain the anticoking effect of Eu doping. The first involves the presence of an additional high-temperature pathway for supplying lattice oxygen based on Eu^3+^ → Eu^2+^ reduction on the surface of the Eu-doped ceria support. Two potential sources of lattice oxygen could prevent carbon deposition on the Ru/CeEuO_2_ catalyst: the Ce^4+^ → Ce^3+^ and Eu^3+^ → Eu^2+^ oxidation state changes on the surface of the ceria support. Our previous studies show that the reductions of Ce^4+^ → Ce^3+^ and Eu^3+^ → Eu^2+^ on the ceria surface in ceria-based catalysts begin at 20–200 °C and 400–600 °C, respectively.^17^ The strong reductive medium of the MSR reaction, combined with the high temperature (700 °C), leads to extensive reduction of Ce^4+^ ions to Ce^3+^ on the surfaces of both (Ru/CeO_2_ and Ru/CeEuO_2_) samples, as confirmed by the XPS data (see Figure S5 and Table S4). Thus, it is reasonable to assume that during the MSR reaction, the Ce^4+^ → Ce^3+^ related pathway for supplying lattice oxygen is more depleted than the Eu^3+^ → Eu^2+^ channel, which may still be active. Meanwhile, analysis of the XPS Eu 3d region indicates that the degree of Eu^3+^ → Eu^2+^ reduction in Ru/CeEuO_2_ is practically the same before and after MSR, at approximately 11% (see Figure S6 and Table S4). Moreover, in our previous work, we found that, despite the similar temperature window of reduction (500–600 °C), Eu_2_O_3_ is much less reducible than CeO_2_.^32^ These observations suggest that the Eu^3+^ → Eu^2+^ related pathway for supplying lattice oxygen is unlikely to be the primary factor responsible for the improved coke resistance of the Ru/CeEuO_2_ catalyst.

Our second hypothesis is that Eu addition to the Ru/ceria catalyst introduces strain into the ceria (support) lattice. It facilitates oxygen diffusion, which in turn results in the prevention of coke deposition on the working Ru/CeEuO_2_ catalyst. This hypothesis is supported by our XRD (see Figure S1 and Table S2) and H_2_-TPR data (see Figure S3 and Table S3). Our third hypothesis suggests that the higher coke resistivity of the Ru/CeEuO_2_ catalyst originates from the Eu-induced modification of the catalyst’s basicity. CO_2_-TPD data collected in Figure S7 show that the Ru/CeEuO_2_ exhibits higher basicity than nondoped Ru/CeO_2_. This aligns well with data by Sato et al., showing that Eu_2_O_3_ has higher basicity than CeO_2_.^22^ Wang et al. reported that an increase in the basicity of Ni-based catalysts enhances their resistance to coking.^25^ Thus, we can tentatively assume that the increase in the catalyst’s basicity is a major factor responsible for the Eu-induced increase in Ru/ceria coking resistance. It is worth noting that the anticoking effect of Eu doping observed in our work requires further verification through in situ experimental techniques and DFT calculations, which we plan to conduct in our next study.

## Conclusions

In this work, we studied the effect of Eu doping on the stability of Ru- and ceria-based catalysts in the MSR reaction for H_2_ production. It was shown that both Ru/CeO_2_ and Ru/CeEuO_2_ catalysts are active in the MSR, with CH_4_ conversion reaching 80–90% at the initial stage of the reaction. However, increasing the reaction time results in a pronounced difference between nondoped and doped catalysts. Specifically, thanks to the far better coking resistance, the Ru/CeEuO_2_ catalyst is significantly more stable than Ru/CeO_2_. Three nonmutually exclusive hypotheses were proposed to explain the anticoking effect of Eu doping. The first is the presence of an extra high-temperature source of lattice oxygen related to Eu^3+^ → Eu^2+^ reduction. The second is that Eu-induced lattice strain facilitates the transport of lattice oxygen to the surface and promotes carbon removal. The third is an increase in the basicity with Eu doping, which in turn inhibits carbon deposition on the working catalyst during the MSR reaction. To validate these hypotheses, detailed investigations using advanced in situ techniques are necessary.

## Supplementary Material



## Notes

Experimental details; chemical composition of the catalysts; XRD patterns and typical TEM images of samples; H_2_-TPR profiles for the as-prepared catalysts; measured by H_2_-TPR and theoretical hydrogen consumption; XPS spectra of Ru 3d + C 1s, Ce 3d, and Eu 3d regions for Ru/CeO_2_ and Ru/CeEuO_2_ samples before and after the MSR reaction; and CO_2_-TPD profiles of Ru/CeO_2_ and Ru/CeEuO_2_ samples (PDF)

## References

[1] Hong
Phuong P., Cam Anh H., Tri N., Phung Anh N., Cam Loc L. (2022). Effect of Support on Stability and Coke Resistance
of Ni-Based Catalyst in Combined Steam and CO2Reforming of CH4. ACS Omega.

[2] Zhang H., Sun Z., Hu Y. H. (2021). Steam Reforming
of Methane: Current States of Catalyst
Design and Process Upgrading. Renewable Sustainable
Energy Rev..

[3] Yang X., Da J., Yu H., Wang H. (2016). Characterization and Performance
Evaluation of Ni-Based Catalysts with Ce Promoter for Methane and
Hydrocarbons Steam Reforming Process. Fuel.

[4] Hu Z., Wang Z., Guo Y., Wang L., Guo Y., Zhang J., Zhan W. (2018). Total Oxidation
of Propane over a
Ru/CeO2 Catalyst at Low Temperature. Environ.
Sci. Technol..

[5] Wang F., He S., Chen H., Wang B., Zheng L., Wei M., Evans D. G., Duan X. (2016). Active Site Dependent Reaction Mechanism
over Ru/CeO2 Catalyst toward CO2Methanation. J. Am. Chem. Soc..

[6] Sorbino G., Di Benedetto A., Italiano C., Thomas M., Vita A., Ruoppolo G., Landi G. (2025). Novel Ni–Ru/CeO2 Catalysts
for Low-Temperature Steam Reforming of Methane. Int. J. Hydrogen Energy.

[7] Trovarelli A., Llorca J. (2017). Ceria Catalysts at
Nanoscale: How Do Crystal Shapes
Shape Catalysis. ACS Catal..

[8] Giordano F., Trovarelli A., De Leitenburg C., Dolcetti G., Giona M. (2001). Some Insight
into the Effects of Oxygen Diffusion in the Reduction Kinetics of
Ceria. Ind. Eng. Chem. Res..

[9] Santos A. C. S. F., Damyanova S., Teixeira G. N. R., Mattos L. V., Noronha F. B., Passos F. B., Bueno J. M. C. (2005). The Effect of Ceria Content on the
Performance of Pt/CeO 2/Al2O3 Catalysts in the Partial Oxidation of
Methane. Appl. Catal., A.

[10] Qin X., Chen X., Chen M., Zhang J., He H., Zhang C. (2021). Highly Efficient Ru/CeO2catalysts
for Formaldehyde Oxidation at Low
Temperature and the Mechanistic Study. Catal.
Sci. Technol..

[11] Tan W., Le S., Diao D., Wang W., Low M., Austin K., Hong D., Gao S., Dong F. (2022). Fine-Tuned
Local Coordination Environment of Pt Single Atoms on Ceria Controls
Catalytic Reactivity. Nat. Commun..

[12] Yang P., Xu J., Tan W., Liu Q., Cai Y., Xie S., Hong S., Gao F., Liu F., Dong L. (2023). Regulating
the Pt1-CeO2 Interaction via Alkali Modification for Boosting the
Catalytic Performance of Single-Atom Catalysts. Chem. Commun..

[13] Araiza D. G., González-Vigi F., Gómez-Cortés A., Arenas-Alatorre J., Díaz G. (2021). Pt-Based Catalysts in the Dry Reforming of Methane:
Effect of Support and Metal Precursor on the Catalytic Stability. J. Mex. Chem. Soc..

[14] Bezkrovnyi O., Kraszkiewicz P., Miśta W., Kepinski L. (2020). The Sintering of Au
Nanoparticles on Flat {100}, {111} and Zigzagged {111}-Nanofacetted
Structures of Ceria and Its Influence on Catalytic Activity in CO
Oxidation and CO PROX. Catal. Lett..

[15] Bezkrovnyi O., Małecka M. A., Lisiecki R., Ostroushko V., Thomas A. G., Gorantla S., Kepinski L. (2018). The Effect of Eu Doping
on the Growth, Structure and Red-Ox Activity of Ceria Nanocubes. CrystEngComm.

[16] Bezkrovnyi O., Szymczak M., Marciniak L., Kraszkiewicz P., Boiko V., Vorochta M., Matolínová I., Kepinski L. (2024). Eu3+ Species as a Luminescent Probe for Fast Monitoring
of the Chemical State of Ceria Catalysts. J.
Phys. Chem. C.

[17] Bezkrovnyi O. S., Mykhailo V., Małgorzata M., Miśta W., Kepinski L. (2020). NAP-XPS Study of Eu3+→Eu2+
and Ce4+→Ce3+
Reduction in Au/ Ce0.80Eu0.20O2 Catalyst. Catal.
Commun..

[18] Amjad U. E. S., Quintero C. W. M., Ercolino G., Italiano C., Vita A., Specchia S. (2019). Methane Steam Reforming
on the Pt/CeO2
Catalyst: Effect of Daily Start-Up and Shut-Down on Long-Term Stability
of the Catalyst. Ind. Eng. Chem. Res..

[19] Salcedo A., Lustemberg P. G., Rui N., Palomino R. M., Liu Z., Nemsak S., Senanayake S. D., Rodriguez J. A., Ganduglia-Pirovano M. V., Irigoyen B. (2021). Reaction Pathway for
Coke-Free Methane Steam Reforming on a Ni/ CeO2Catalyst: Active Sites
and the Role of Metal-Support Interactions. ACS Catal..

[20] Baudh A., Garjola M., Sharma R., Sharma S., Upadhyay R. K. (2024). Effect
of Ceria Morphology on Hydrogen Production via Methane Steam Reforming
for Membrane Reformer. Can. J. Chem. Eng..

[21] Summa P., Samojeden B., Motak M. (2019). Dry and Steam Reforming of Methane.
Comparison and Analysis of Recently Investigated Catalytic Materials.
A Short Review. Pol. J. Chem. Technol..

[22] Sato S., Takahashi R., Kobune M., Gotoh H. (2009). Basic Properties of
Rare Earth Oxides. Appl. Catal., A.

[23] Anh A. N., Nguyen H. H., Pham T. P., Pham L. K. H., Ha
Le Phuong D., Nguyen N. A., Vo D. V., Pham P. T. H. (2023). Insight
into the Role of Material Basicity in the Coke Formation and Performance
of Ni/Al2O3 Catalyst for the Simulated- Biogas Dry Reforming. J. Energy Inst..

[24] Sirous
Rezaei P., Shafaghat H., Daud W. M. A. W. (2015). Suppression
of Coke Formation and Enhancement of Aromatic Hydrocarbon Production
in Catalytic Fast Pyrolysis of Cellulose over Different Zeolites:
Effects of Pore Structure and Acidity. RSC Adv..

[25] Wang S., Lu M. (1998). Catalytic Activities
and Coking Characteristics of Oxides-Supported
Ni Catalysts for CH4 Reforming with Carbon Dioxide. Energy Fuels.

[26] Bezkrovnyi O., Kraszkiewicz P., Vorochta M. (2024). In Situ Study of the
Effect of the
Exposed Surface of Ceria (100 vs 111) on the Highly Oxidized Species
Formation on Ru/Ceria Catalysts. Acta Phys.
Polym., A.

[27] Bezkrovnyi O., Vorokhta M., Pawlyta M., Ptak M., Piliai L., Xie X., Dinhová T. N., Khalakhan I., Matolínová I., Kepinski L. (2022). In Situ Observation
of Highly Oxidized Ru Species in Ru/CeO2 Catalyst under Propane Oxidation. J. Mater. Chem. A.

[28] Schilling C., Hofmann A., Hess C., Ganduglia-Pirovano M. V. (2017). Raman Spectra
of Polycrystalline CeO2: A Density Functional Theory Study. J. Phys. Chem. C.

[29] Huang H., Dai Q., Wang X. (2014). Morphology
Effect of Ru/CeO2 Catalysts for the Catalytic
Combustion of Chlorobenzene. Appl. Catal., B.

[30] Binnemans K. (2015). Interpretation
of Europium­(III) Spectra. Coord. Chem. Rev..

[31] Wu J., Lin M. L., Cong X., Liu H. N., Tan P. H. (2018). Raman Spectroscopy
of Graphene-Based Materials and Its Applications in Related Devices. Chem. Soc. Rev..

[32] Bezkrovnyi O. (2025). Comprehensive
H2-TPR Study of the Lanthanide Oxides Reducibility. J. Cluster Sci..

