# Supplementary material for: Anticoking Effect of Eu3+ Doping of the Ru/Ceria Catalyst in the MSR Reaction for Hydrogen Generation
Source: J Phys Chem C Nanomater Interfaces. 2025 Oct 3;129(41):18513–8. doi: 10.1021/acs.jpcc.5c05324 (PMC12536495; doi:10.1021/acs.jpcc.5c05324)
Supplement: Supplementary file 1 [file jp5c05324_si_001.pdf]

Electronic Support Information (ESI)

Anti-coking Effect of Eu^{3+} Doping of Ru/Ceria Catalyst in MSR Reaction for Hydrogen Generation

Oleksii Bezkrovnyi[1*], Núria J. Divins[2,3], Isabel Serrano[2], Xènia Garcia[2,3], Piotr Kraszkiewicz[1], Maciej Ptak[1], Mirosława Pawlyta,[4,5] Leszek Kępiński[1] and Jordi Llorca[2,3]

[1] W. Trzebiatowski Institute of Low Temperature and Structure Research, Polish Academy of Sciences, 50-422 Wroclaw, Poland

[2] Institute of Energy Technologies, Universitat Politècnica de Catalunya, EEBE, 08019 Barcelona, Spain

[3] Department of Chemical Engineering and Center for Research in Multiscale Science and Engineering, Universitat Politècnica de Catalunya, EEBE, 08019 Barcelona, Spain

[4] School of Chemistry, University of StAndrews, St Andrews KY16 9ST, United Kingdom

[5]MaterialsResearch Laboratory, Silesian University of Technology, Gliwice 44-100, Poland

*Corresponding author: o.bezkrovnyi@intibs.pl

Experimental details.

Synthesis procedure. CeO_2 and $Ce_{0.80}Eu_{0.20}O_2$ supports were synthesized by simple thermal decomposition of nitrates. Stoichiometric amounts of $Ce(NO_3)_3{\cdot}6H_2O$ and $Eu(NO_3)_3{\cdot}6H_2O$ (POCH, ≥99.9 % purity) were dissolved in 10 ml of deionized water and stirred for 30 min at room temperature to obtain a homogeneous solution. The solution was then transferred to a porcelain crucible, dried at 60 °C for 12 h, and calcined in air at 700 °C for 3 h. Next, 800 mg of the support was ultrasonically dispersed in 50 ml H_2O. Then, an appropriate amount of ruthenium (III) nitrosylnitrate solution containing 11wt % Ru was added to the ceria suspension to get 2.5 wt% Ru catalyst, which was ultrasonically treated for 30 min. Next, a few drops of ammonia solution (30% in water) were added to increase the pH from 1.5 to 9.5, and the suspension was stirred for 30 min. The obtained suspensions of Ru/CeO_2 or $Ru/Ce_{0.80}Eu_{0.20}O_2$ catalysts were centrifuged. Precipitates were washed five times with deionized H_2O, dried at 60 °C for 12 h, and annealed in H_2 at 700 °C for 3 h. In the following, these samples are referred to as Ru/CeO_2 and $Ru/CeEuO_2$.

Catalysts characterization. The crystal structure of the samples was determined by powder X-ray diffraction (XRD) using an X'Pert PRO PANalytical diffractometer with Cu Kα radiation. Calculations of lattice parameters, average crystallite size, and average strain were performed by Rietveld refinement using the FullProf programme [1] (version November 2023).

The Thompson-Cox-Hastings (TCH) pseudo-Voigt function was applied to fit the diffraction profiles, enabling the estimation of size and strain broadening contributions.

The morphology of the samples was determined by scanning electron microscopy (SEM, FEI Nova NanoSEM 230) and transmission electron microscopy (TEM, Philips CM-20 SuperTwin instrument operating at 160 kV). Raman spectra (100-4000 cm^{-1}) were measured using a Renishaw InVia Raman spectrometer equipped with a confocal DM 2500 Leica optical microscope, a thermoelectrically cooled CCD as a detector, and an argon laser operating at 514.5 nm. Each spectrum was collected three times with an acquisition time of 20 s, using the 20×/0.4 microscope magnification lens. The chemical composition of the samples was verified by energy dispersive X-ray spectroscopy (EDS) using a FEI Nova NanoSEM 230 instrument equipped with an EDAX Genesis XM4 detector. The surface of the catalysts was studied by X-ray photoelectron spectroscopy (XPS) using a laboratory SPECS system with a PHOIBOS 150 EP Hemispherical Energy Analyzer and a MCD-9 detector. Data processing was performed with the CasaXPS program (Casa Software Ltd., UK). The reducibility of the as-received samples was studied by H_2-TPR (temperature-programmed reduction), performed by heating the samples at a heating rate of 10 °C/min up to 1000 °C in H_2/Ar (5 vol%) flow (30 ml/min). The hydrogen consumption was monitored by a thermal conductivity detector (TCD). CO_2-temperature programmed desorption (CO_2-TPD) were performed using a Micromeritics AutoChem II 2920 apparatus equipped with MS. Before the H_2-TPR test, the samples (20 mg) were pretreated with 5%H_2/Ar (30 cm^3/min) at 500 °C for 30 min, cooled down to 30 °C and purged by He flow (30 cm^3/min). Next, the samples were exposed on pure CO_2 flow (30 cm^3/min) for 60 min. The CO_2-TPD experiment was performed under heating up to 500 °C at 10 °C/min in He flow (30 cm^3/min).

Catalytic Test Details. MSR tests were performed by mixing the catalysts with SiC in quartz reactors (fixed bed reactor, inner diameter 8 mm) at atmospheric pressure. Catalysts were activated in 10% H_2/N_2 (50 mL min^{-1}) from room temperature to 300 °C (10 °C min^{-1}), keeping this temperature for 1 hour. After the reduction, the MSR mixtures consisted of I) 30 mL/min N_2, 20 mL/min CH_4, and 40 mL/min H_2O; and II) 60 mL/min N_2, 40 mL/min CH_4, and 80 mL/min H_2O was dosed. Water was injected with an HPLC pump (Knauer) and vaporized at 120 °C. The catalytic activity was investigated at 700 °C using 100 mg of samples diluted with SiC to a total bed volume of 1 mL. For these experiments, a flow-to-weight ratio (F/W) = 54 000 and 108 000 mL $gcat^{-1}h^{-1}$ and space velocity (GHSV) = 389 879 and 779 758 h^{-1} were chosen.

The reaction products were analyzed using a micro-GC (Agilent Technologies 3000A Micro GC) equipped with a molecular sieve 5Å.

Table S1. Chemical composition of the Ru/CeO_2 and $Ru/CeEuO_2$ catalysts.

Sample	Support's formula		Ru content, wt.%	
	nominal	EDS	nominal	EDS
Ru/CeO_2	CeO_2	CeO_2	2.50	2.40
$Ru/CeEuO_2$	$Ce_{0.8}Eu_{0.2}O_2$	$Ce_{0.81}Eu_{0.19}O_2$	2.50	2.61

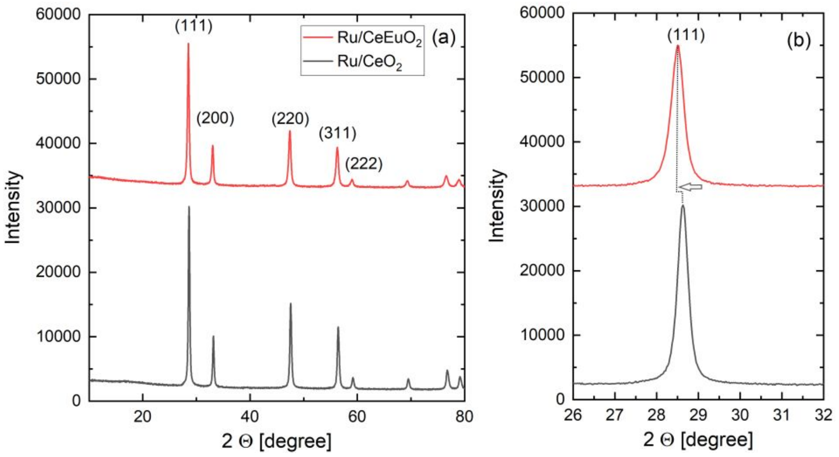

Fig. S1. Powder XRD patterns of the as-received Ru/CeO_2 and $Ru/CeEuO_2$ samples.

Table S2 Structural parameters of Ru/CeO_2 and $Ru/CeEuO_2$ samples calculated using FullProf.

Sample	Average cryst. size, [nm]	Average strain $*10^{-4}$	Unit cell parameters						
			a, [nm]	b, [nm]	c, [nm]	α, [°]	β, [°]	γ, [°]	Space group
Ru/CeO_2	31.08 +/-0.02	16.46 +/- 0.01	0.54128 +/- 0.00005	0.54128	0.54128	90	90	90	*Fm*-3*m*
$Ru/CeEuO_2$	36.00 +/-0.02	52.05 +/- 0.02	0.54218 +/- 0.00008	0.54218	0.54218	90	90	90	*Fm*-3*m*

Note: The standard deviations appearing in the global average apparent size and strain are calculated using the different reciprocal lattice directions. It is a measure of the degree of anisotropy, not of the estimated error.

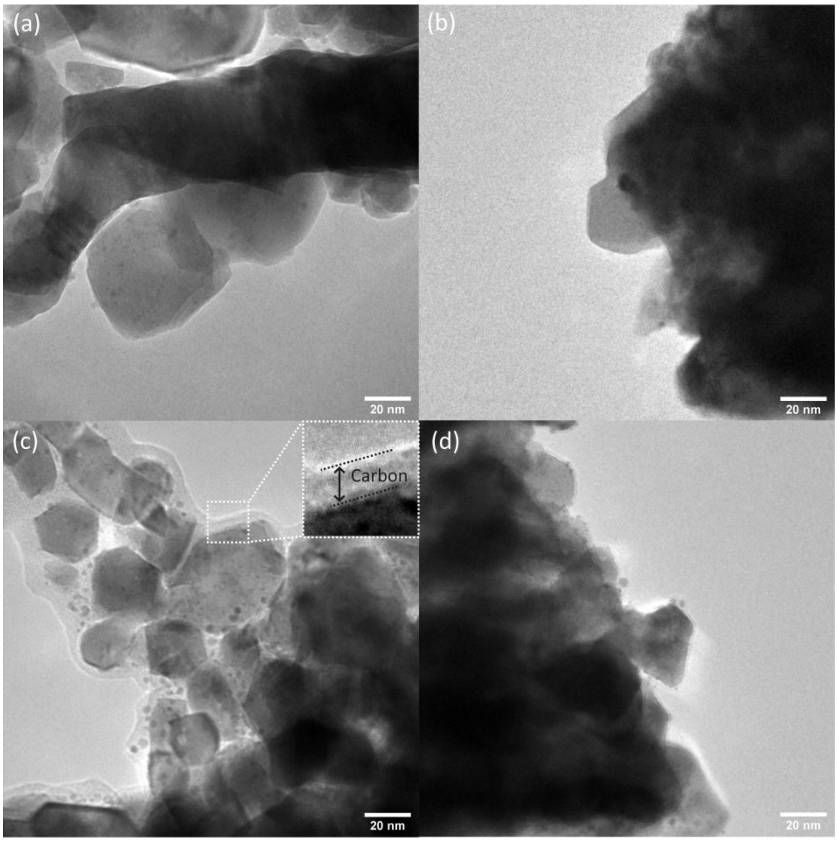

Fig. S2. Typical TEM images of Ru/CeO_2 and $Ru/CeEuO_2$ catalysts before (a and b) and after (c and d) MSR reaction.

As shown in Figs. S2c-d, the amount of carbon deposited on Ru/CeO_2 catalysts after the MSR reaction is significantly greater than that on the $Ru/CeEuO_2$. The carbon deposit on the Ru/CeO_2 catalyst (Fig. S2c) is continuous and 5-10 nm thick. On the contrary, a much thinner layer (~1-2 nm) occurs on $Ru/CeEuO_2$ (Fig. S2d). However, taking into account the local character of TEM, we used TEM imaging as an additional technique to complement the Raman data (which we used as the primary tool to estimate the degree of C accumulation).

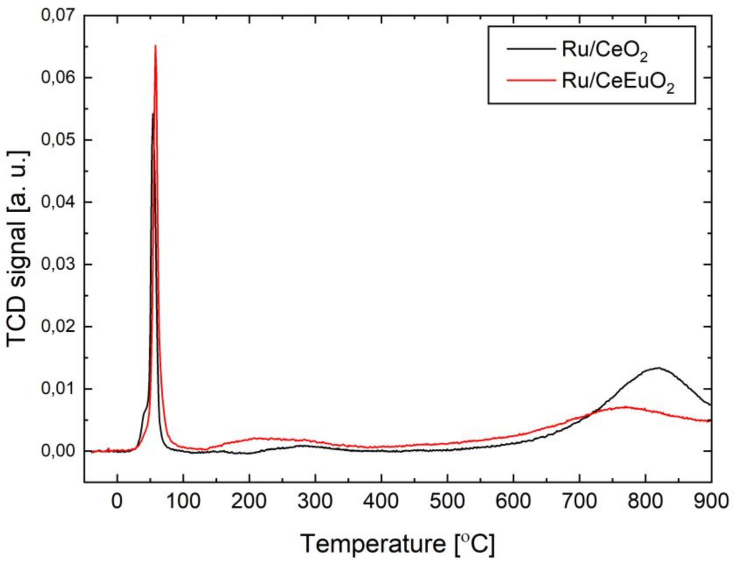

Fig. S3. H_2-TPR curves for the as-prepared catalysts.

Table S3. Measured by H_2-TPR and theoretical hydrogen consumption.

Sample	H_2 consumption [μmol/$g_{cat.}$] – measured				H_2 consumption [μmol/g_{cat}] – theoretical[a]		
	0–100 °C	0–300 °C	0–600 °C	0–900 °C	RuO_2	Support	Catalyst (total)
Ru/CeO_2	182	189.2	218.2	902.8	494	2 810	3 304
$Ru/CeEuO_2$	217.2	302.6	414.6	898.4	494	2 238	2 732

[a] Assuming total reduction of RuO_2, total reduction of Ce^{4+} to Ce^{3+}, and no reduction of Eu^{3+}, for a fully oxidized catalyst.

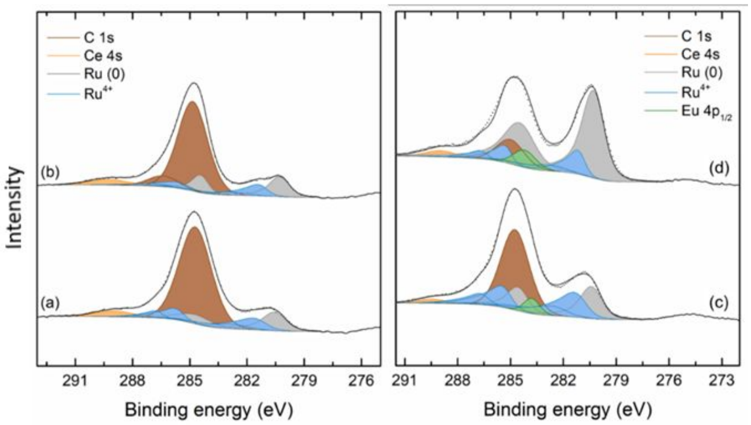

Fig. S4. XPS spectra of Ru3d+C1s region for Ru/CeO_2 and $Ru/CeEuO_2$ catalysts before (a and c) and after MSR reaction (b and d)

Deconvolution of ruthenium 3d spectra was based on the peak positions reported in the literature.[2-5] They were fitted with two peaks for Ru^0 (one doublet) and four peaks for RuO_2 (two doublets).

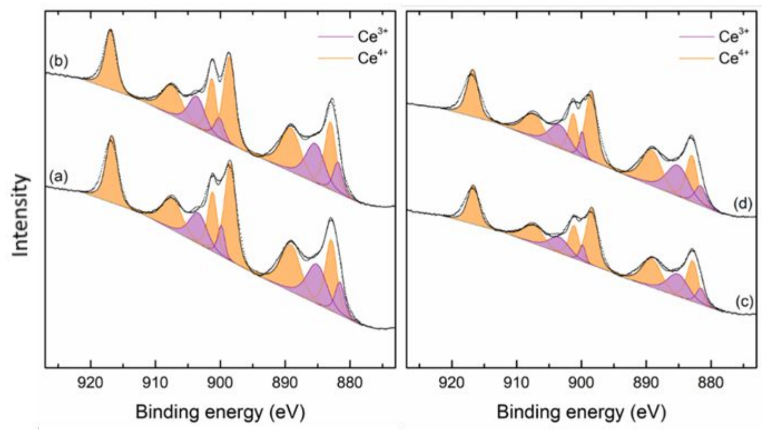

Fig. S5 XPS spectra of Ce3d region for Ru/CeO_2 and Ru/$CeEuO_2$ catalysts before (a and c, respectively) and after MSR reaction (b and d, respectively)

As reported in the literature,[6] six peaks for Ce^{4+} (V, V'', V''', U, U'' and U'''), corresponding to three pairs of spin-orbit doublets, along with four peaks for Ce^{3+} (V_0, V', U_0 and U'), corresponding to two doublets, were employed for cerium 3d spectra. U and V refer to the $3d_{3/2}$ and $3d_{5/2}$ spin-orbit components, respectively. Spectra have been normalized with respect to the U''' peak of cerium and to the adventitious carbon peak in the case of Ru 3d.

Eu3d region was fitted with two doublets at (1135 eV (Eu^{3+} $3d_{3/2}$) and 1165 eV (Eu^{3+} $3d_{5/2}$)) and (1125 eV (Eu^{2+} $3d_{3/2}$) and 1135 eV (Eu^{2+} $3d_{5/2}$)) related to Eu^{3+} and Eu^{2+}, respectively.[7]

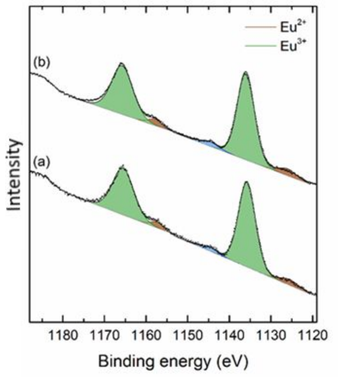

Fig. S6. XPS spectra of the Eu3d region recorded for Ru/$CeEuO_2$ sample before (a) and after MSR reaction (b). Blue peaks around 1144 eV are assigned to shake-down satellites of Eu $3d_{5/2}$ main peak.

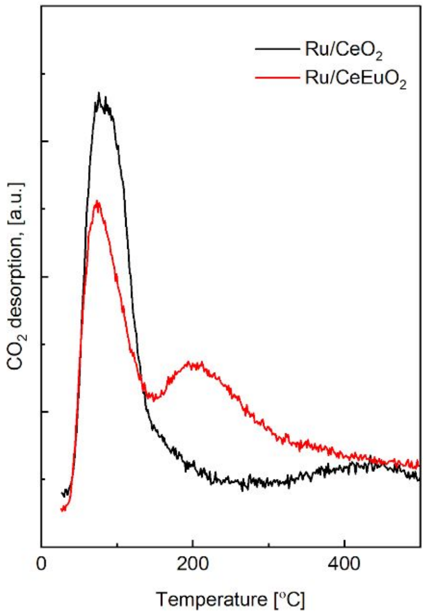

Fig. S7. The CO_2-TPD patterns of Ru/CeO_2 and Ru/$CeEuO_2$ samples.

As shown in Fig. S7, the undoped Ru/CeO_2 sample exhibits a single CO_2-TPD peak centered at 100 °C. Adding the Eu to ceria support leads to a second wide peak formation at 200 °C, which suggests the presence of strong basic sites absent on the undoped sample.

Table S4 Summarizing table of XPS data.

Sample	Ce^{4+}percantege [%] (Ce3d region)	Eu^{2+}percantege [%] (Eu3d region)	Ru^0 percentage [%] (Ru3d region+C1s region)
Ru/CeO_2	72	-	45
Ru/CeO_2 after MSR	71	-	52
Ru/$CeEuO_2$	73	10	40
Ru/$CeEuO_2$ after MSR	69	12	80

References:

1. Rodriguez-Carvajal, J (1993). Recent advances in magnetic structure determination by neutron powder diffraction. Physica B., 192, 55 – 69. https://doi.org/10.1016/0921-4526(93)90108-I
2. Morgan, D.J. (2015). Resolving ruthenium: XPS studies of common ruthenium materials. Surf. Interface Anal. 47, 1072–1079. https://doi.org/10.1002/sia.5852
3. Matienzo, J., Yin, L.I., Grim, S.O., and Swartz, W.E. (1973). X-Ray Photoelectron Spectroscopy of Nickel Compounds. Inorg. Chem. 12, 2762–2769. https://doi.org/10.1021/ic50130a005
4. Bianchi, C.L., Ragaini, V., and Cattania, M.G. (1991). An XPS study on ruthenium compounds and catalysts. Mater. Chem. Phys. 29, 297–306
5. Elmasides, C., Kondarides, D.I., Gru¨ nert, W., and Verykios, X.E. (1999). XPS and FTIR Study of Ru/Al 2 O3 and Ru/TiO 2 Catalysts: Reduction Characteristics and Interaction with a MethaneOxygen Mixture. J. Phys. Chem. B 103, 5227–5239. https://doi.org/10.1021/jp9842291
6. Mullins, D.R., Overbury, S.H., and Huntley, D.R. (1998). Electron spectroscopy of single crystal and polycrystalline cerium oxide surfaces. Surf. Sci. 409, 307–319. https://doi.org/10.1016/S0039-6028(98)00257-X
7. Kang, J. S.; Jeong, Y. K.; Kang, J. G.; Zhao, L.; Sohn, Y.; Pradhan, D.; Leung, K. T. Observation of Mediated Cascade Energy Transfer in Europium-Doped ZnO Nanowalls by 1,10-Phenanthroline. Journal of Physical Chemistry C 2015, 119 (4), 2142–2147. https://doi.org/10.1021/jp5090795.
